# Could blocking the formation of amyloid channels rescue Alzheimer's phenotype?

**DOI:** 10.15252/emmm.201708491

**Published:** 2017-12-05

**Authors:** Francesc X Guix, Carlos G Dotti

**Affiliations:** ^1^ Centro de Biología Molecular Severo Ochoa CSIC/UAM Madrid Spain

**Keywords:** Chromatin, Epigenetics, Genomics & Functional Genomics, Neuroscience, Pharmacology & Drug Discovery

## Abstract

In a most simplified way, we can say that much of the symptomatology that characterizes Alzheimer's disease (AD) can be attributed to a cascade of toxic events initiated by the presence in the interstitial space of the brain of oligomers of the β‐amyloid peptide (Aβ) peptide, a cleavage by‐product of the Amyloid precursor protein (APP). Intuitively, it follows that the amyloid peptide is the ideal target to combat this disease. However, several anti‐Aβ therapies failed in clinical trials devoted to find a treatment for AD. However, last year, the results of a clinical trial prompted back the interests in this type of therapy. In this issue of *EMBO Molecular Medicine*, Martinez Hernandez and colleagues present encouraging results showing that the diphenylpyrazole compound Anle138b prevents and reduces the toxic effects of Aβ in a mouse model of AD (APPPS1ΔE9). Regarding the mechanisms of action, they present good evidence that Anle138b prevents the formation of conducting Aβ pores on artificial membranes and primary hippocampal neurons. While the data are encouraging, AD mouse models only represent part of the AD pathology and clinical trials are needed to determine the usefulness of Anle138b to treat AD patients.

Alzheimer's disease is a devastating neurodegenerative disorder affecting millions of people around the world and the first cause of dementia. According to the amyloid cascade hypothesis (Selkoe & Hardy, 2016), the deposition in the brain of Aβ in form of amyloid plaques triggers a series of downstream pathological events that lead to the intraneuronal aggregation of the microtubule‐stabilizing protein tau in form of neurofibrillary tangles (NFT) and neural degeneration (for a more comprehensive, and less simplistic, view please see De Strooper & Karran, 2016). Consequently with this line of thought, several lines of therapeutic interventions have been suggested and assayed in clinical trials, ranging from anti‐aggregating compounds or antibodies against amyloid plaques and NFT to the inhibition of the proteases responsible for Aβ generation or anti‐neuroinflammatory agents fetching the preservation of the integrity of synaptic structures and their function (reviewed in Selkoe & Hardy, 2016). However, up to the present moment, none of these treatments have successfully passed phase III when assayed in clinical trials that included mild‐to‐moderate AD patients (reviewed in Sala Frigerio & De Strooper, 2016). One of the most recurrent arguments to explain such failures is that the interventions are initiated too late in the disease process and early preventive treatments would provide benefits. The problem resides on the identification of those people at risk of developing AD in order to start a preventive therapy before the onset of the symptoms. Studies carried out in familial AD, an early‐onset form of the disease caused by dominant mutations on the genes responsible for Aβ generation, have highlighted the existence of a large prodromal phase where biochemical alterations in the cerebrospinal fluid (CSF) and morphological changes of the brain detected by PET are observed up to 10 years before the appearance of the first symptoms (Bateman *et al*, 2012). This opens a window for therapeutic intervention devoted to prevent the cognitive decline. However, PET is expensive and limited in availability, and CSF sampling is unpopular with patients. Due to the lack of inexpensive and non‐invasive reliable methods to identify people at risk for AD, we are currently limited to the treatment of patients once the clinical symptoms are evident.

Despite the lack of success of the clinical trials aimed to slow down the cognitive decline in AD, a recent study using a monoclonal antibody (Aducanumab) against the Aβ peptide showed encouraging results and prompted back interest in drugs targeting neurotoxic Aβ aggregates. AD patients who received Aducanumab had a decrease in the number of amyloid plaques observed by PET and a slower cognitive decline (Sevigny *et al*, 2016), suggesting that therapeutic interventions targeting Aβ could be beneficial. The new study of Martinez Hernandez *et al* (2018) in the current issue of *EMBO Molecular Medicine* present solid evidence that the diphenylpyrazole compound Anle138b prevents and reduces the toxic effects of Aβ in a mouse model of AD, giving new hope that attacking Aβ can produce therapeutic benefits. Anle138b was first described as an anti‐oligomeric agent capable of blocking in different mouse models the neurotoxic effects of tau, prion, and α‐synuclein oligomers, which are associated to human tauopathies, prion, and Parkinson's diseases, respectively (Matthes *et al*, 2017). Martinez Hernandez *et al* (2018) now show that Anle138b also interferes with Aβ toxicity. They first observed that Anle138b was able to improve survival times in a *Drosophila* model of amyloid‐induced neurotoxicity. This result encouraged the authors to analyze the effect in an AD mouse model (APPPS1ΔE9). This AD mouse model starts to show Aβ deposits by 6 months of age, and by 8–10 months of age, some neuronal loss is observed around amyloid plaques. By now, we are all well aware that there is no such thing as a “mouse” model for AD and that the current models show only some features of the disease, most typically aberrant production of Aβ and/or deposition of amyloid plaques. This is the case in the mouse line utilized and therefore one should refrain from being too enthusiastic about the observations. However, the results obtained are most promising and deserve careful follow‐up. In short, what Martinez Hernandez and colleagues found in this mouse line is that a 4‐month treatment with Anle138b improved long‐term potentiation (LTP) in the hippocampus, the cellular response to learning and memory processes. Consistently, the Anle138b treatment was paralleled by an improved spatial memory in the Morris water maze test, in comparison with the impaired spatial memory seen in animals with the same genetic background but treated with a placebo. Anle138b only enhanced memory in the AD mouse model but did not affect memory consolidation in wt animals. Interestingly enough, the above‐mentioned results were observed in mice at both pre‐plaque (2‐month‐old) and post‐plaque (6‐month‐old) stages, suggesting that Anle138b is an efficient amyloid‐induced toxicity inhibitor.

In order to elucidate how Anle138b might be operating, Martinez Hernandez and colleagues performed a series of different experimental approximations. They first compared by RNA‐sequencing the pathways affected in the hippocampus between wild‐type (wt) and APPPS1ΔE9 mice at the pre‐plaque stage and found several alterations in genes (203 genes) related to cell growth, energy metabolism, mitochondrial function, cytoskeleton, and synaptic plasticity. Interestingly, most of these alterations were reverted by the administration of Anle138b, suggesting that the overall effect of these gene variations interfere with function (remember: the Anle138b treatment improves both LTP and learning). However, in the post‐plaque scenario, they found a lower number of deregulated genes (130 genes) in the APPPS1ΔE9 mice compared to the wt, and 113 of those genes, which are mostly associated to neuroinflammatory processes, remained unchanged after administration of Anle138b. The fact that the pathways deregulated at the pre‐plaque and the post‐plaque stages are not the same could suggest, among other possibilities, that the first response of cells to the presence of the toxic peptide is aimed at guaranteeing survival (at the expense of performance?) while the second response, due to the lasting presence of the noxious peptide, is to orient the cells toward irreversible dysfunction and eventually death. This could account for the alteration of neuroinflammatory pathways in 10‐month‐old APPPS1ΔE9 mice. However, neuroinflammation is not reduced by Anle138b treatment even when this treatment improved synaptic plasticity, implying that the severity of the phenotype was not due to the activation of these genes (the treatment rescues phenotype but not the miss‐expression of neuroinflammation genes). Quite interestingly, this is not the case in the murine tauopathy model TAUP301S. Neuroinflammation‐related genes are affected in this line and Anle138b treatment restores the wt condition. These apparently contradictory results suggested to the authors of this study that Anle138b is not a specific inhibitor of any given “toxic” pathway but that it probably works through blocking upstream initiators of the pathology that in each model or pathological stage triggers neurotoxicity by different pathways.

Indeed, it is believed that several neurodegenerative diseases, including AD, are initiated by the formation of neurotoxic oligomers (Walsh & Selkoe, 2007). Anle138b has been shown to inhibit the formation of oligomers formed by several disease‐associated proteins (Matthes *et al*, 2017). In order to evaluate whether part of the effect of Anle138b is caused by the interference of Aβ aggregation, Martinez Hernandez *et al* (2018) analyzed brain sections of APPPS1ΔE9 mice by thioflavin staining which had been previously treated with placebo or Anle138b at the pre‐plaque or post‐plaque stage. The number and the area of amyloid plaques were lower in Anle138b‐treated mice; however, the effect was more pronounced in the pre‐plaque group. Although these results would strongly suggest that Anle138b blocks Aβ aggregation and by this prevents toxicity, it is currently thought that Aβ oligomers and not amyloid plaques are responsible for neurotoxicity (Walsh & Selkoe, 2007). In light of this possibility, the authors tested whether Anle138b beneficial effects occurred by interfering with Aβ‐formed pores. This idea arises from the hypothesis, formulated by Arispe *et al* (1993), that Aβ oligomers form Ca^2+^‐selective pores on the plasma membrane of neurons and induce neural death by increasing the permeability to Ca^2+^. Martinez Hernandez *et al* (2018) have tested the ability of Anle138b to prevent Aβ pores from destabilizing biological membranes by measuring *in vitro* the conductivity of artificial lipidic membranes (pore‐free membranes do not conduct the current) composed of the lipids POPE and DOPS. While Aβ oligomers increased the conductivity of these artificial membranes, treatment with Anle138b blocked this effect (Fig 1). Interestingly, the blockage was achieved without affecting the number of Aβ pores or their surface structure on the artificial membranes determined by Atomic Force Microscopy. Instead, the authors suggest that Anle138b triggers a conformational change in Aβ pores that turn them into being non‐conductive (Fig 1). This effect was also observed on primary hippocampal neurons, where decreased membrane integrity caused by Aβ oligomers was restored by Anle138b.

**Figure 1:**
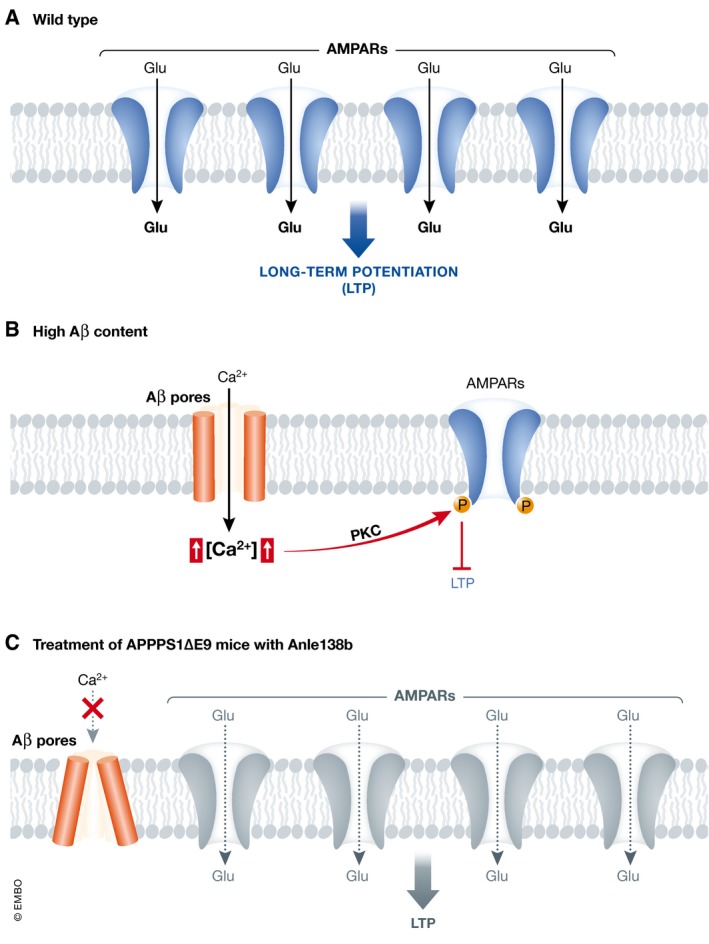
Inhibition of amyloid pore‐induced toxicity by Anle138b Mechanism of action for Anle138b. (A) In wt mice, high‐frequency stimulation or spike‐timed stimulation induces LTP, a biological process behind memory formation. In a most simplified way, the calcium influx induced by the stimulation drives the insertion of AMPA receptors required for LTP stabilization. (B) In conditions with a high Aβ content in the interstitial space (i.e., the synaptic cleft), as could be the case in APPPS1ΔE9 mice, Aβ oligomers form calcium‐selective pores in the plasma membrane of neurons, allowing a massive influx of calcium cations into the cytosol. The sudden and exaggerate increase in the concentration of cytosolic calcium now plays a LTP‐blocking role, perhaps by decreasing the number of AMPA receptors inserted on the surface of synapses by the PKC‐dependent phosphorylation on serine 880 of the AMPA subunit GluR2 (Liu *et al*, 2010). (C) The treatment of APPPS1ΔE9 mice with Anle138b induces a conformational change of the Aβ pores, turning them into non‐conductive and preventing in this way the detrimental downstream effects on LTP and memory formation. Although the authors do not report how the blockage of Aβ pores restores LTP, the reduction in calcium influx suggests that it might well be through the normalization of AMPA receptor insertion at the synaptic membrane.

The mechanism proposed by Martinez Hernandez *et al* (2018), to explain the beneficial effect of Anle138b, is well supported by the data, and may be relevant in AD. It is in fact possible that the beneficial effect attributed to the conformational/conductance changes on the Aβ pores could also explain previous results showing that Aβ oligomer leads to cell toxicity by interfering neurotransmitter–receptor function, through a change in plasma membrane organization and fluidity.

In conclusion, the study carried out by Martinez Hernandez *et al* (2018) show that Anle138b may be a potential drug to treat the amyloid pathology in AD patients even when clinical symptoms are already present. Due to the fact that Anle138b has been shown to delay disease progression in a mouse model of tauopathy, it could provide an extra‐beneficial effect on AD patients by targeting the NFT lesions. Only very reasonably is the team cautiously extrapolating their results to humans, since AD models only represent a part of the complex AD pathology. Only clinical trials will tell whether Anle138b could become a successful therapeutic option.
